# Capturing Translation in Action with Protein Synthesis Profiling

**DOI:** 10.1101/2025.11.17.688896

**Published:** 2025-11-17

**Authors:** Cesar Arcasi Matta, Zhi Qi Ten, Simpson Joseph

**Affiliations:** Department of Chemistry and Biochemistry, University of California at San Diego, 9500 Gilman Drive, La Jolla, CA 92093-0314 USA

## Abstract

Translation is a central control point of gene expression, linking nucleotide sequences to functional proteins. Dysregulated translation contributes to diverse diseases, underscoring the need for methods that can directly reveal which transcripts are actively translated. Ribosome profiling, the current gold standard, provides nucleotide-resolution maps of ribosome occupancy but requires laborious purification and sacrifices information on mRNA isoforms and mRNA modifications by restricting analysis to short ribosome-protected fragments.

Here, we introduce Protein Synthesis Profiling (PSP), a proximity-labeling strategy for transcriptome-wide identification of actively translated mRNAs without ribosome isolation. PSP exploits a fusion of the enzyme APEX2 with the elongation factor eEF2, which transiently associates with ribosomes during elongation, to catalyze selective tagging of mRNAs engaged in translation.

Applied in *Saccharomyces cerevisiae*, PSP captures condition-specific translational programs, recapitulates known stress responses, and expands the detectable repertoire of regulated genes beyond existing methods. By preserving full-length transcript features, PSP is scalable, isoform-aware, and broadly adaptable, providing a versatile platform to dissect translational regulation in health and disease.

## Introduction

Dysregulation of mRNA translation contributes to a wide spectrum of human diseases and metabolic syndromes^1,2^. Despite its importance, a comprehensive understanding of which mRNAs are actively translated in healthy versus diseased states remains incomplete. Ribosome profiling is the gold-standard method for measuring translation in vivo, offering nucleotide-resolution maps of ribosome occupancy^3,4^. Ribosome profiling begins by halting mRNA translation, treating the mRNA-ribosome complex with nuclease to generate ribosome footprints, and then recovering the ribosome by ultracentrifugation^4^. Following ultracentrifugation, the ~30-nucleotide ribosome-protected fragments are purified and put through library preparation for deep sequencing^4^. Although ribosome profiling is commonly used, it has several limitations and challenges. First, it is a laborious technique requiring large material quantities and sophisticated instruments^4^. Second, preparing sequencing libraries from the ~30-nt ribosome-protected fragments may introduce experimental bias and contaminating footprints, which contribute to mapping ambiguous reads^5^. Third, there is a loss of information regarding mRNA isoforms and post-transcriptional modifications because the short ribosome-protected fragments are not compatible with long-read sequencing methods.

Proximity labeling is a robust method to identify proteins and RNAs localized near a protein of interest within living cells^6^. This technique utilizes engineered enzymes fused to query proteins to generate reactive species that covalently label nearby biomolecules in vivo. Among these enzymes, APEX2, an optimized ascorbate peroxidase, efficiently converts biotin-tyramide into reactive radicals with a short half-life (<1 ms), tagging biomolecules within ~25 nm^7–13^. Importantly, APEX-seq has demonstrated that APEX2 fusion proteins can effectively label and identify RNAs in living cells through streptavidin enrichment and deep sequencing^11,12,14,15^. Leveraging APEX-seq, we established Protein Synthesis Profiling (PSP), a novel chemical tagging approach that enables transcriptome-wide identification of actively translated full-length mRNAs without the need for fragmentation. PSP utilizes a fusion of ascorbate peroxidase 2 (APEX2) with eukaryotic elongation factor 2 (eEF2), which transiently interacts with ribosomes during the elongation cycle of protein synthesis. Upon activation, APEX2-eEF2 tags nearby molecules, selectively labeling mRNAs engaged in active translation. As a proof of concept, we established PSP in *Saccharomyces cerevisiae*, where it effectively enriched for condition-specific translated transcripts.

## Results

### Rational design of the APEX2-eEF2 fusion for ribosome-proximal labeling

**Figure 1.**
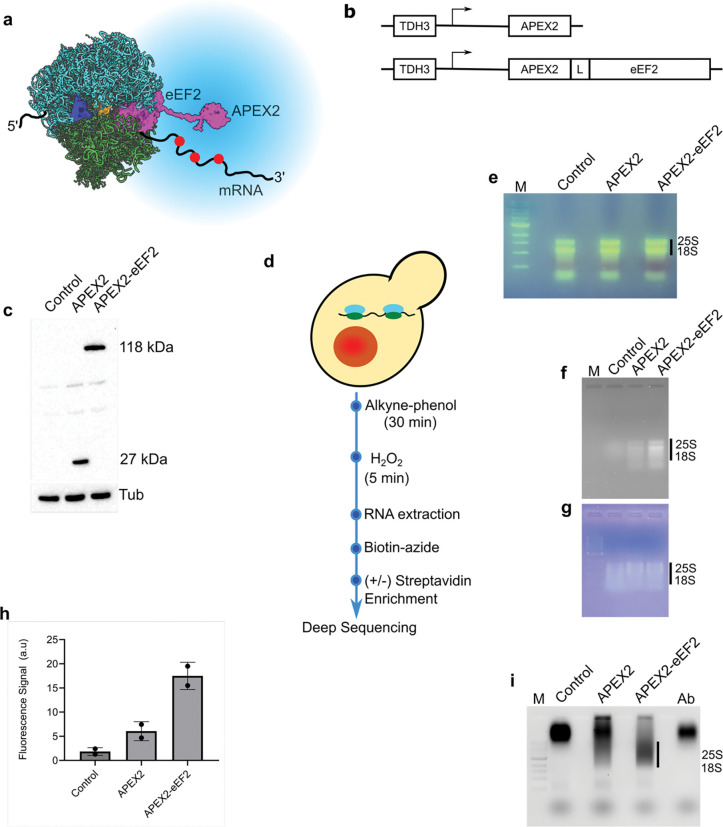
Design and validation of APEX2-eEF2 for RNA tagging in yeast. **a,** Structural model of APEX2-eEF2 bound to the ribosome. The large ribosomal subunit is shown in cyan, the small subunit in green, APEX2-eEF2 in magenta, and mRNA as a black line with the red circles indicating the alkyne modification. The blue sphere marks the ~25 nm labeling radius of APEX2. **b,** Expression constructs for APEX2 and APEX2-eEF2 under the TDH3 promoter. **c,** Western blot detection of APEX2 and APEX2-eEF2 expression. Lanes: control (empty vector), APEX2 (27 kDa), and APEX2-eEF2 (118 kDa). Tubulin (Tub) was probed as a loading control. **d,** Schematic of the RNA tagging workflow. Yeast cells were incubated with alkyne-phenol (30 min), followed by H_2_O_2_ (5 min). After quenching, total RNA was extracted, conjugated to biotin-azide via click chemistry, and enriched with streptavidin beads. Both total and enriched RNA were used for preparing Illumina sequencing libraries. **e,** Agarose gel analysis of total RNA from control, APEX2, and APEX2-eEF2 cells. The presence of intact 25S and 18S rRNA bands indicates high RNA quality. M, molecular weight ladder. **f,** Detection of alkyne-labeled RNAs by conjugation with fluorescein-azide. Total RNA from control, APEX2, and APEX2-eEF2 cells was subjected to click chemistry and analyzed by agarose gel electrophoresis. Fluorescence was detected using a Typhoon imager. **g,** The same gel as in F, stained with SafeStain to verify equal RNA loading. **h,** Quantification of fluorescein-labeled RNA signal. The bar graph shows fluorescence intensity normalized to total RNA, averaged across two independent experiments. **i,** Gel-shift assay of biotin-labeled RNAs incubated with anti-biotin-AF488 antibody. RNAs from control, APEX2, and APEX2-eEF2 cells were conjugated with biotin-azide, bound by antibody, and resolved on an agarose gel. Antibody-RNA complexes are indicated by the black bar. Lanes: M, molecular weight ladder; control, RNA from control cells; APEX2, RNA from APEX2-expressing cells; APEX2-eEF2, RNA from APEX2-eEF2-expressing cells; Ab, antibody only.

We engineered the APEX2-eEF2 fusion protein by analyzing the structural organization of eEF2 and its interaction with the 80S ribosome during mRNA-tRNA translocation. To identify the optimal fusion site, we examined the high-resolution X-ray crystal structure of eEF2 bound to the eukaryotic 80S ribosome from *S. cerevisiae* (Figure 1a)^16^. APEX2 was fused to the N-terminus of eEF2, positioned away from the decoding center and the mRNA-tRNA complex to avoid disrupting translation. This site is advantageous because domains I and II of eEF2 maintain a stable spatial association with the ribosome, whereas domains III-V undergo conformational changes necessary for translocation^16^. To further minimize interference with GTP hydrolysis or translocation, a flexible 31-amino acid linker was inserted between APEX2 and eEF2. Structural modeling with AlphaFold^17^ indicated that ribosome-bound mRNAs remain within a ~25 nm radius of the APEX2-eEF2 fusion, enabling efficient labeling.

For in vivo implementation, the APEX2-eEF2 fusion gene was placed under the control of the strong, constitutive *TDH3* promoter (encoding glyceraldehyde-3-phosphate dehydrogenase) (Figure 1b) and integrated into the chromosome of *S. cerevisiae* strain ZY10^18^. In parallel, a control strain was constructed in which APEX2 alone was expressed from the same *TDH3* promoter. This TDH3-APEX2 strain served as a background control to distinguish nonspecific RNA labeling by APEX2 from ribosome-associated tagging.

### Enhanced ribosome-proximal RNA labeling with APEX2-eEF2

We examined the expression of APEX2 and the APEX2-eEF2 fusion protein by Western blotting (Figure 1c). A FLAG epitope was present at the N-terminus of APEX2, allowing detection with an anti-FLAG antibody. As expected, the strain expressing APEX2 displayed a band at ~27 kDa, while the strain expressing the APEX2-eEF2 fusion protein showed a band at ~118 kDa, consistent with the predicted molecular weights of the respective proteins. No FLAG-reactive band was detected in the control strain lacking APEX2 or APEX2-eEF2 expression. Together, these results confirm the successful in vivo expression of both APEX2 and the APEX2-eEF2 fusion protein.

A previous study demonstrated that alkyne-phenol penetrates the yeast cell wall more efficiently than biotin-phenol, resulting in improved APEX2 labeling^19,20^. To perform proximity RNA labeling, control, APEX2, and APEX2-eEF2 yeast strains were grown to mid-log phase, treated with alkyne-phenol for 30 minutes, and then exposed to hydrogen peroxide for 5 minutes to initiate labeling (Figure 1d). Total RNA was purified and analyzed by agarose gel electrophoresis, which showed intact, high-quality 25S and 18S rRNAs (Figure 1e).

To determine whether the purified RNAs were tagged with alkyne groups by APEX2, we performed copper-catalyzed alkyne-azide click chemistry using a 6-fluorescein-azide (FAM-azide) dye on aliquots of total RNA from each strain. The FAM-labeled RNAs were purified, normalized for concentration, resolved on an agarose gel, and imaged on a Typhoon scanner (Figure 1f). Both APEX2 and APEX2-eEF2 samples showed fluorescent RNA bands compared to the negative control, with the APEX2-eEF2 strain exhibiting ~3-fold higher fluorescence intensity than APEX2 alone (Figure 1f, h). The major fluorescent bands corresponded to 25S and 18S rRNAs, with a diffuse smear below the 18S rRNA consistent with labeled mRNAs. Parallel RNA staining with a non-specific dye confirmed equal RNA loading across samples (Figure 1g). Together, these results indicate that APEX2 non-specifically tags a low fraction of total cellular RNAs (~5%), whereas APEX2-eEF2 achieves enhanced RNA labeling (~15%), likely due to its association with translating ribosomes.

We next tested whether alkyne-tagged RNAs could be conjugated to biotin. Total RNA from control, APEX2, and APEX2-eEF2 strains was reacted with biotin-picolyl-azide via click chemistry, followed by RNA purification. The biotinylated RNAs were then incubated with an anti-biotin antibody conjugated to Alexa Fluor 488. The RNA-antibody mixtures were separated by agarose gel electrophoresis and visualized on a Typhoon imager (Figure 1i).

The antibody alone migrated above the 10 kb marker, and a similar band was detected with control RNA. In the APEX2 sample, we primarily observed this free antibody band along with a faint smear extending toward the 25S and 18S rRNAs (near the 3 kb and 2 kb markers). In contrast, the APEX2-eEF2 RNA sample displayed a markedly stronger fluorescent signal corresponding to the rRNAs. This result parallels the direct RNA labeling experiment with FAM-azide, where APEX2-eEF2 showed enhanced fluorescence compared to APEX2. Together, these findings demonstrate that APEX2-eEF2 preferentially tags ribosome-associated RNAs above the background labeling observed with APEX2 alone, further supporting its functional utility for selective RNA labeling on the ribosome.

### Transcriptional and translational reprogramming under amino acid starvation

To validate that the APEX2-eEF2 fusion protein proximity labels ribosomes and potentially ribosome-bound mRNAs, we performed amino acid starvation experiments in yeast. The transcriptional and translational responses to acute amino acid deprivation are well characterized in *S. cerevisiae* and therefore provide a robust benchmark^3,21^. Yeast expressing APEX2-eEF2 was grown to mid-log phase and then divided into two aliquots. One aliquot was transferred to complete medium as the control, while the other was transferred to medium lacking amino acids for 20 minutes to induce starvation. Both cultures were subsequently treated with alkyne-phenol and hydrogen peroxide to initiate APEX2-catalyzed proximity labeling.

Total RNA was extracted from control and amino acid-starved cells and conjugated to biotin-azide. An aliquot of the biotinylated RNA from each condition was enriched with streptavidin beads to isolate RNAs preferentially tagged because of their ribosome association. Illumina sequencing libraries were then prepared from (i) total RNA (before streptavidin enrichment) and (ii) ribosome-associated RNA (after enrichment) for both control and amino acid-starved conditions. In addition, a reference library was generated from total RNA extracted from yeast grown to mid-log phase without any treatment, providing a baseline transcriptome.

For each condition, four independent biological replicates were performed and used to generate Illumina libraries for deep sequencing. Sequencing data quality was first assessed with FastQC, and reads were aligned to the *S. cerevisiae* reference genome using STAR^22^, followed by indexing and sorting with Samtools^23^. On average, ~80 million paired-end reads were obtained per sample, with >70% mapping uniquely to the yeast genome. Gene-level read counts were assigned using FeatureCounts^24^. Sample quality was further evaluated with a variance-stabilizing transformation (VST) in DESeq2^25^ (Figure S1a - b). The VST-normalized expression data were analyzed by principal component analysis (PCA), which confirmed replicate concordance, revealed minimal variability within conditions, and identified no extreme outliers across experimental groups or sequencing batches (Figure S1c-d). PCA also facilitated detection of potential batch effects, characterization of biological variation (e.g., amino acid-rich vs. amino acid-deprived samples), and comparison of transcriptional versus ribosome-associated RNA profiles. As expected, the principal source of variance distinguished RNAs from amino acid-deprived cells versus control cells, confirming that amino acid starvation induces global shifts in gene expression.

### Differential translational control revealed by PSP

To benchmark PSP, we compared our dataset with two landmark studies of amino acid deprivation in *S. cerevisiae*: the ribosome profiling analysis by Ingolia et al.^3^ and the polysome profiling dataset by Smirnova et al.^21^ Differential gene expression analysis using Anota2seq was performed to characterize both transcriptional and translational responses to amino acid starvation^26–29^. We used the nominal p-values for the comparative evaluation between Anota2seq and the log_2_-ratio method used by Ingolia et al.^3^ and Smirnova et al.^21^ to ensure a fair, rank-based contrast of detection performance. Anota2seq models the dependency between total and translated RNA levels to identify genes subject to distinct modes of post-transcriptional regulation. Total RNA from control and starved yeast was compared with the corresponding streptavidin-enriched (translated) RNA under identical conditions. This analysis classified genes into three principal categories: (1) Translationally regulated, showing significant changes in translation efficiency independent of mRNA abundance; (2) mRNA abundance, driven primarily by altered transcript levels; and (3) Translationally buffered, in which opposing transcriptional and translational changes maintain constant protein output^26–29^.

**Figure 2.**
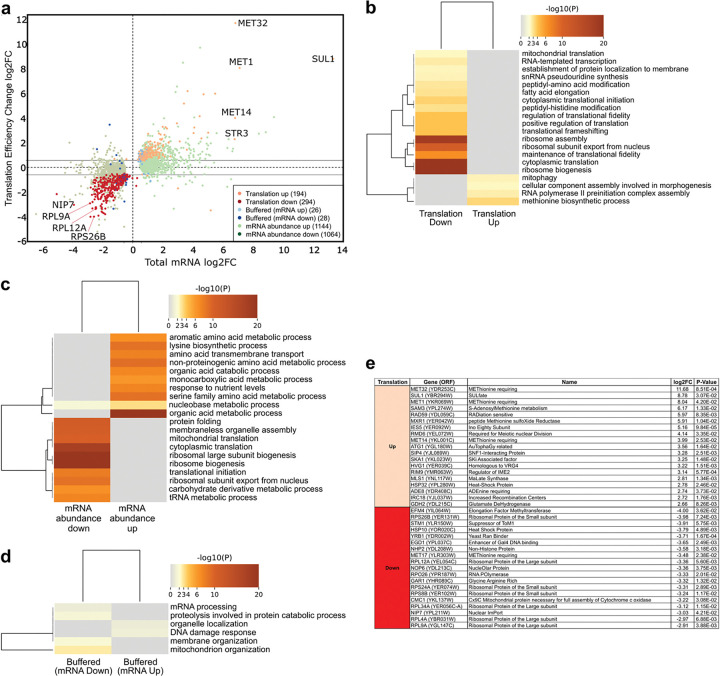
Transcriptional and translational responses to amino acid starvation. **a,** Scatter plot comparing log_2_ fold change (FC) in total mRNA and translation efficiency between control and amino acid-starved cells. Genes were classified by anota2seq into three regulatory categories: translation, buffered, and mRNA abundance, which are color-coded as indicated. **b – d,** Heat maps showing Gene Ontology (GO) enrichment for the major regulatory classes: **b,** translationally downregulated and upregulated, **c,** change in mRNA abundance, and **d,** buffered mRNA downregulated and upregulated. Rows correspond to enriched biological processes and columns to the individual datasets; colors denote −log_10_ (P) values. **e,** Representative examples of the most significantly affected genes with a large change in translation efficiency and their annotated functions.

Amino acid starvation induced extensive reprogramming of both transcription and translation. In total, 194 genes were translationally upregulated and 294 translationally downregulated (Figure 2a). Gene Ontology (GO) analysis^30^ of the translationally upregulated group revealed enrichment for methionine biosynthesis, RNA polymerase II-mediated transcription, cellular morphogenesis, and mitophagy (Figure 2b). These pathways reflect a nutrient-scavenging program that enhances amino-acid uptake, recycling, and de novo synthesis to preserve intracellular amino-acid homeostasis through coordinated activation of autophagy, transport, and anabolic metabolism.

In contrast, translationally downregulated genes were strongly enriched for ribosome biogenesis, cytoplasmic translation, ribosomal assembly, and subunit export (Figure 2b). This widespread repression of ribosome-related functions likely represents a resource-conservation strategy that suppresses energetically costly protein synthesis during nutrient limitation.

At the mRNA abundance level, starvation triggered broad changes, with 1,144 genes upregulated and 1,046 downregulated. Upregulated genes were associated with amino acid metabolism, nutrient signaling, and amino acid transport, whereas downregulated genes mirrored translational repression, encompassing ribosome biogenesis, cytoplasmic translation, and mitochondrial translation (Figure 2c). These parallel trends underscore the coordinated downshift in global biosynthetic capacity.

Finally, 54 genes displayed translational buffering, including 26 transcriptionally upregulated and 28 transcriptionally downregulated transcripts whose translation levels remained stable. Buffered upregulated genes were enriched for DNA-damage response and organelle localization, suggesting a transient uncoupling of translation from mRNA accumulation to preserve proteostasis, while buffered downregulated genes - linked to mitochondrial organization and mRNA processing - may enable rapid translational reactivation upon nutrient recovery (Figure 2d).

### PSP recapitulates and extends known starvation responses

At the gene level, PSP analysis revealed that amino acid starvation selectively enhances translation of MET32, SUL1, MET1, SAM3, MXR1, MET14, and STR3, core regulators and enzymes of the methionine and sulfur assimilation network (Figure 2e). Their coordinated induction promotes sulfate import and methionine biosynthesis, complementing the well-characterized Gcn2-Gcn4 transcriptional program^31,32^ and uncovering an additional translational layer that sustains sulfur amino acid homeostasis. PSP also detected increased translation of RAD59, IES5, ATG1, and HSP32, implicating elevated DNA repair capacity, chromatin remodeling, autophagy initiation, and protein quality control as auxiliary stress-mitigation pathways (Figure 2e).

Conversely, translation of EFM4, RPS26B, STM1, RPL12A, NOP6, RPS24A, RPS8B, RPL34A, NIP7, RPL4A, and RPL9A was strongly repressed (Figure 2e), consistent with the conserved energy-saving response that curtails ribosome biogenesis and protein synthesis under nutrient limitation^33–35^. Notably, several of these genes encode ribosomal proteins or assembly factors whose starvation-induced repression was not fully resolved by previous approaches. Together, these findings demonstrate that PSP robustly captures the canonical starvation program while unveiling previously obscured layers of translational regulation that fine-tune metabolic reallocation and proteome remodeling during nutrient stress.

**Figure 3.**
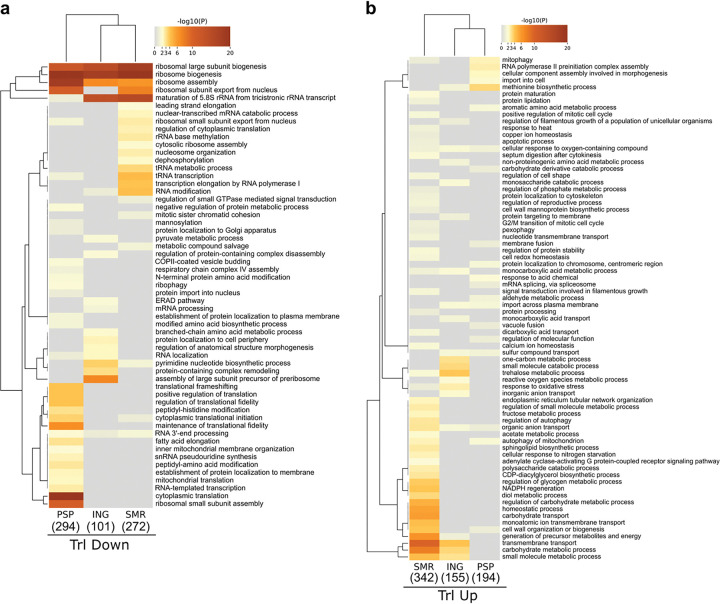
Comparative functional analysis of PSP, ribosome profiling, and polysome profiling under amino acid starvation. **a – b,** Heat maps showing GO term enrichment for **a,**translationally downregulated and **b,** translationally upregulated genes identified by PSP, Ingolia *et al.* (ING), and Smirnova *et al.* (SMR). Rows correspond to enriched biological processes and columns to the individual datasets; colors denote −log_10_(P) values.

To evaluate concordance with existing methods, we benchmarked PSP against the Ingolia (ING)^3^ and Smirnova (SMR)^21^ datasets. ING identified 155 translationally upregulated and 101 downregulated genes, while SMR reported 342 and 272, respectively. Gene Ontology (GO) enrichment analysis across all three datasets revealed striking thematic agreement (Figs. 3a - b). Among downregulated GO terms, each method showed strong enrichment for ribosome biogenesis and assembly, confirming repression of ribosomal pathways as a conserved starvation response (Fig. 3a). PSP, however, uniquely resolved additional repressed categories, including cytoplasmic translation (e.g., SUI2, NIP1, RPS3), translational fidelity (e.g., ASC1, RPS31, RPL3), and programmed ribosomal frameshifting (e.g., HYP2, ANB1, SSB2). These processes reflect active modulation of ribosome function and decoding accuracy, key hallmarks of translational economy during amino acid scarcity^33–37^.

Conversely, the GO terms associated with upregulated genes across PSP, ING, and SMR datasets were enriched for import across the plasma membrane, sulfur compound transport, and methionine biosynthetic process, reflecting a coordinated amino acid-starvation response that promotes sulfur assimilation and nutrient uptake to sustain translation (Figure 3b). Notably, PSP uniquely uncovered additional induced categories, including aromatic amino acid metabolic process (e.g., TRP4, HIS7, BNA6), import into cell (e.g., MUP1, ZRT2, ECM21), and carbohydrate derivative catabolic process (e.g., PED1, APA2, YND1). These PSP-specific enrichments point to an expanded translational control program that integrates amino acid biosynthesis, nutrient import, and carbon mobilization - hallmarks of the adaptive metabolic reprogramming mediated by the Gcn2/GCN4 axis under amino acid limitation^29,37–42^.

Collectively, these findings demonstrate that PSP not only reproduces the canonical translational signatures of amino acid starvation but also extends them by uncovering finer layers of control affecting ribosome function and metabolic remodeling.

### PSP shows partial gene-level overlap but strong global concordance

**Figure 4.**
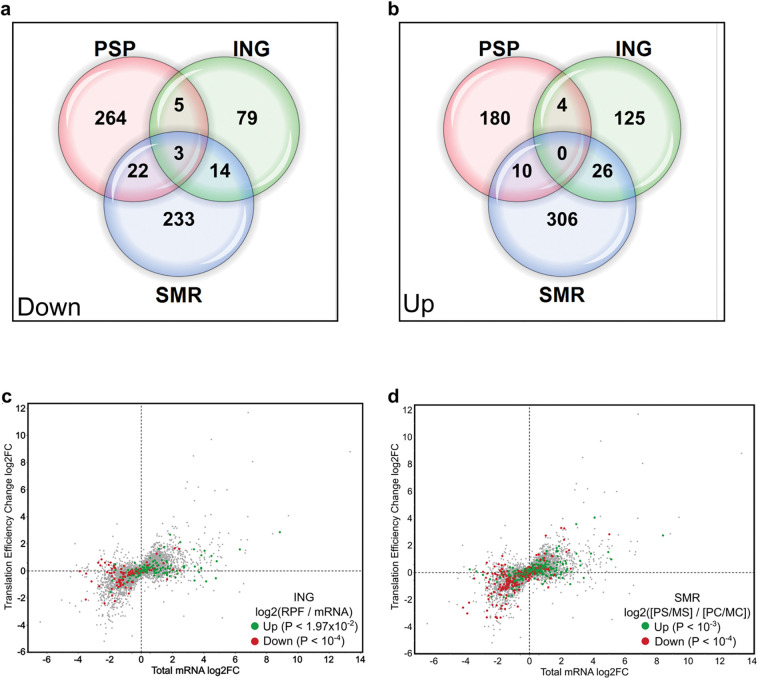
Gene-level overlap and global concordance among PSP, ribosome profiling, and polysome profiling datasets. **a – b,** Venn diagrams illustrating the overlap of differentially translated genes identified by PSP, ING, and SMR. **c,** Scatter plot comparing PSP data (grey, plotted as in Fig. 2a) with ING data overlaid for translationally downregulated (red) and upregulated (green) genes. Significance was determined by a one-sided Mann-Whitney U test (*P* values shown). **d,** Scatter plot comparing PSP data (grey, plotted as in Fig. 2a) with SMR data overlaid for translationally downregulated (red) and upregulated (green) genes. Significance was determined by a one-sided Mann-Whitney U test (*P* values shown).

Gene-specific overlap analysis revealed limited intersection among datasets. Of the 294 PSP-downregulated genes, 22 overlapped with SMR only, 5 with ING only, and 3 were shared across all three datasets (Fig. 4a); 14 genes were common to SMR and ING. Among the 194 PSP-upregulated genes, 10 overlapped with SMR only, 4 with ING only, and 26 were shared between SMR and ING (Fig. 4b). Such modest overlap is characteristic of high-throughput expression studies and likely reflects differences in experimental platforms, sequencing depth, and analytical models. Consistent with this observation, Larsson *et al.* reanalyzed the ING ribosome profiling data and reported that the traditional log_2_-ratio method used by Ingolia *et al.* identified largely non-overlapping sets of translationally regulated genes compared to those detected by the statistically rigorous Anota2seq approach^29^. Thus, the limited overlap at the gene-specific level is not unexpected given the distinct analytical frameworks employed; importantly, the GO analysis reveals strong concordance in the underlying pathways that are translationally regulated across datasets.

Despite this gene-level divergence, a one-way Mann-Whitney U test revealed strong global concordance between PSP and both ING and SMR (Figs. 4c-d; *P* < 10^−2^–10^−4^). Some transcripts that appeared translationally regulated in ING or SMR exhibited minimal change in PSP, consistent with Anota2seq’s capacity to account for covariance between total and translated RNA. By explicitly separating transcriptional from translational effects, PSP provides a more accurate estimate of translational control.

Together, these analyses confirm that PSP faithfully reproduces the conserved starvation-induced programs of translational repression and metabolic remodeling, while extending their resolution to additional regulatory layers. By directly coupling translation-linked RNA labeling with deep sequencing, PSP delivers a mechanistic and high-fidelity view of how cells remodel translation in response to nutrient stress.

## Discussion

Translation is the decisive checkpoint between RNA and protein, yet it remains the most challenging layer of gene expression to study at scale. Ribosome profiling delivers nucleotide resolution but fragments transcripts and requires labor-intensive purification, while polysome profiling provides only coarse estimates of translational activity^4,43,44^. To overcome these limitations, we developed Protein Synthesis Profiling (PSP), a proximity-labeling approach that directly marks ribosome-associated RNAs in vivo. By fusing APEX2 to eEF2, a factor that transiently engages ribosomes during elongation, PSP selectively labels mRNAs in the act of translation while preserving their full length. This unique feature makes PSP compatible with long-read sequencing and enables the study of isoforms, untranslated regions, and RNA modifications that are inaccessible to footprint-based methods.

Our proof-of-concept in *S. cerevisiae* demonstrates that PSP can detect both global and gene-specific changes in translation under nutrient stress. In response to amino acid starvation, PSP faithfully captured the canonical translational reprogramming response, downregulation of ribosome biogenesis and translation factor mRNAs coupled with upregulation of amino acid biosynthesis, amino acid transport, and autophagy pathways. These findings align closely with results from ribosome profiling and polysome profiling^3,21^. Importantly, PSP identified a broader spectrum of regulated genes than either traditional method, which we attribute to deeper sequencing coverage and preservation of transcript integrity. This expanded coverage highlights PSP’s ability to detect subtle and previously underappreciated translational changes, providing a more complete view of the adaptive gene expression program.

Equally significant are the methodological advantages of PSP. The workflow is streamlined and scalable, avoiding labor-intensive fractionation steps and reducing technical variability, which makes the approach broadly accessible. Furthermore, PSP is versatile and adaptable, readily applicable to diverse experimental designs, stress conditions, and biological systems, and can be transferred to mammalian cells without major technical barriers.

Looking ahead, PSP has the potential to reshape the study of translational control. By directly labeling ribosome-associated RNAs in vivo, PSP provides a framework that can be extended to dynamic studies of translation in response to environmental stress, signaling pathways, developmental programs, and disease states. The method is also inherently compatible with emerging single-cell and spatial transcriptomics platforms, offering a path to high-resolution mapping of translational regulation across heterogeneous tissues, developmental gradients, and tumor microenvironments^45–47^. This ability to bridge transcriptional, translational, and spatial layers of gene regulation represents a unique strength of PSP.

In summary, PSP expands the experimental toolkit for investigating translational regulation by combining biochemical specificity with methodological simplicity. Its enhanced transcript coverage, broad adaptability, and compatibility with cutting-edge sequencing technologies position PSP not merely as a complement to ribosome and polysome profiling, but as a next-generation standard for probing translation in complex biological systems. By lowering technical barriers while increasing biological insight, PSP opens new opportunities to study translation with unprecedented depth, breadth, and resolution - poised to have a transformative impact on molecular biology, systems biology, and biomedical research.

Finally, the clinical implications of PSP are far-reaching. Many human diseases, including cancer, neurodegeneration, and metabolic disorders, are driven by aberrant translational control^1,2^. By enabling transcriptome-wide analysis of actively translated RNAs in vivo and in intact tissues, PSP provides a practical and scalable strategy to dissect disease-associated translational programs. Its compatibility with clinical samples, single-cell technologies, and spatial mapping suggests that PSP could eventually serve as a powerful diagnostic and therapeutic discovery tool, bridging basic mechanistic insight with translational medicine.

## Materials and Methods

### Plasmid construction and yeast transformation

The pTDH3-APEX2 plasmid was generated by PCR amplification of the *APEX2* gene from pJH124 (Addgene #102951) and insertion into p406TDH3 (Addgene #15977) by homologous recombination. To create pTDH3-APEX2-eEF2, the yeast *eEF2* gene was PCR-amplified from pTKB612 (a gift from Dr. Jonathan D. Dinman, University of Maryland) and inserted into pTDH3-APEX2 by homologous recombination. All constructs were verified by whole-plasmid sequencing (Plasmidsaurus).

The pTDH3-APEX2 and pTDH3-APEX2-eEF2 plasmids were linearized with *NcoI-HF* and transformed into *S. cerevisiae* strain ZY10 (W303 background: MATa trp1 leu2 ura3 his3 can1 GAL+ psi+)^18^ (a gift from Dr. Brian Zid, UCSD) to achieve chromosomal integration. Transformations were carried out using the Frozen-EZ Yeast Transformation II kit (Zymo Research, Cat# T2001) according to the manufacturer’s protocol. Chromosomal integration was confirmed by PCR and Sanger sequencing.

### Protein extraction and Western blotting

A single yeast colony from a Synthetic Complete (SC) (-Ura) agar plate having 2% D-glucose was inoculated into 3 mL of SC (-Ura) medium with 2% D-glucose and grown overnight at 30 °C. The culture was diluted to an OD600 of ~0.1 in 10 mL of fresh SC (-Ura) medium with 2% D-glucose and incubated at 30 °C until the OD600 reached ~1.0. Cells were harvested by centrifugation at 4,835 × g for 15 minutes at 4 °C, and the pellet was washed twice with 5 mL 1× PBS (pH 7.4). The washed pellet was resuspended in 200 μL of 0.2 M NaOH, incubated at room temperature for 5 minutes, and centrifuged at 12,000 rpm for 1 minute. The pellet was resuspended in 100 μL of 2× SDS loading buffer (24 mM Tris-HCl pH 6.8, 10% glycerol, 0.8% SDS, 5.76 mM β-mercaptoethanol, 0.04% bromophenol blue) and boiled at 95 °C for 5 minutes. Lysates were centrifuged at 3,000 rpm for 10 minutes at 4 °C, and supernatants were collected.

Protein concentration was estimated by A280 measurement using a Spark Multimode Microplate Reader (Tecan, Switzerland). For SDS-PAGE, 2.5 μg of total protein was loaded onto a 10% gel alongside a pre-stained protein ladder (NEB, Cat# P7706). Electrophoresis was performed in two steps: 100 V for 10 minutes, followed by 180 V for 40 minutes. Proteins were transferred onto a PVDF membrane pre-activated in methanol for 30 seconds, rinsed in deionized water (5×), and equilibrated in transfer buffer (25 mM Tris, 192 mM glycine, 20% methanol) for 15 minutes. Transfer was performed using a Bio-Rad Trans-Blot SD semi-dry system at 20 V for 1 hour.

Membrane was blocked in 5% milk for 1 hour at room temperature with gentle shaking, washed 5 times with 1× TBST (20 mM Tris, 150 mM NaCl, 0.1% Tween 20), and incubated overnight at 4 °C with mouse anti-FLAG Tag monoclonal antibody (1:5,000 dilution) (Invitrogen Cat# MA1–91878) in 5% milk. After washing 3 times with 1× TBST, the membrane was incubated with HRP-conjugated anti-mouse IgG secondary antibody (1:10,000 dilution) (Cell Signaling Technology Cat# 7076) for 1 hour at room temperature. After additional washes, chemiluminescence was developed with Amersham ECL substrate and imaged using a Bio-Rad ChemiDoc MP Imaging System.

To check for loading control, the membrane was stripped in stripping buffer (10 mM β-mercaptoethanol, 62.6 mM Tris-HCl pH 6.8, 2% SDS) at 55 °C for 40 minutes. The blot was reprobed with mouse anti-alpha-tubulin monoclonal antibody (1:8000 dilution) (DSHB Cat# 12G10) in 5% milk for 2 hours at room temperature, followed by the HRP-conjugated anti-mouse IgG secondary antibody (1:10,000 dilution) and detected as described above.

### APEX2-catalyzed alkyne-phenol tagging of RNA

Yeast cells were cultured in 60 mL of SC (-Ura) media with 2% D-glucose at 30 °C with vigorous shaking. Cultures were inoculated at an initial OD600 of 0.1 and grown to mid-log phase (OD600 = 0.6–0.7). Cells were harvested, washed once with 1× PBS (pH 7.4), and resuspended in 500 μL of 1× PBS supplemented with 2.5 mM alkyne-phenol (MedChemExpress LLC, Cat# HY-131442). The suspension was incubated at room temperature with constant shaking for 30 minutes.

To initiate APEX2 labeling, 5 μL of 100 mM hydrogen peroxide was added, briefly mixed by gentle vortexing, and incubated at room temperature for 5 minutes. The reaction was quenched by adding 500 μL of quenching buffer (10 mM NaN_3_, 5 mM Trolox, 10 mM sodium ascorbate in 1× PBS), followed by gentle vortexing. Cells were pelleted by centrifugation at 15,294 × g for 5 minutes at 4 °C, and the supernatant was discarded. The pellet was washed twice with 500 μL of 1× PBS and resuspended in FAE solution (98% formamide, 10 mM EDTA, pH 8).

The suspension was heated at 70 °C for 10 minutes, then centrifuged at 21,000 × g for 2 minutes at room temperature. The RNA-containing FAE supernatant was transferred to a fresh DNA/RNA Lo-Bind 1.5 mL microcentrifuge tube, diluted to 78% formamide with water, and purified using the RNA Clean & Concentrator-100 kit (Zymo Research), according to the manufacturer’s instructions.

The concentration of alkyne-labeled RNA was measured with a Spark Multimode Microplate Reader (Tecan, Switzerland), and RNA quality was assessed by 1% TAE agarose gel electrophoresis. DNase treatment was performed using RQ1 RNase-Free DNase (Promega, Cat# M6101) following the manufacturer’s protocol. DNase-treated RNA was further purified using the Monarch RNA Cleanup Kit (New England BioLabs, Cat# T2040). Final RNA concentration was determined using the microplate reader, quality was verified by agarose gel electrophoresis, and samples were stored at −80 °C.

### Click chemistry labeling of RNA

A total of 100 μg of alkyne-labeled RNA was used for the click reaction. The reaction mixture contained 10 mM Tris-HCl (pH 7.5), 2 mM biotin picolyl azide (Click Chemistry Tools, Cat# 1167), 0.1 mM CuSO_4_, 2 mM THPTA, and 10 mM sodium ascorbate. Samples were vortexed, briefly centrifuged, and incubated in the dark at room temperature for 30 minutes with constant shaking.

After incubation, RNA was purified using the Monarch RNA Cleanup Kit (New England BioLabs, Cat# T2040) according to the manufacturer’s instructions. The RNA concentration was determined with a Spark Multimode Microplate Reader (Tecan, Switzerland), integrity was assessed by 1% TAE agarose gel electrophoresis, and samples were stored at −80 °C.

### Fluorescein labeling of RNA

A total of 20 μg of alkyne-labeled RNA was subjected to a click reaction with 2 mM 6-fluorescein-azide (Jena Bioscience, Cat# CLK-80105), as described above. From this reaction, 3 μg of fluorescein-labeled RNA was mixed with 1× loading dye and resolved on a 1% TAE agarose gel at 70 V for 40 minutes. Alternatively, 5 μg of fluorescein-labeled RNA was analyzed by 10% urea-PAGE.

In-gel fluorescence was detected using an Amersham Typhoon Biomolecular Imager (Cytiva, MA, USA) with Cy2 configuration (488 nm laser, 525BP20 filter, 50 μm pixel size). Fluorescence intensity of dye-labeled RNA was quantified using the BandPeak tool in ImageJ. Following fluorescence imaging, the agarose gel was stained in 30 mL of 1× TAE buffer containing 3 μL SYBR Gold nucleic acid gel stain (Invitrogen) for 1 hour with gentle shaking and visualized under UV illumination to assess total RNA.

### Detection of biotinylated RNA

A total of 10 μg of biotinylated RNA in 1× PBS was incubated with 250 ng of anti-biotin monoclonal antibody (BK-1/39) conjugated to Alexa Fluor 488 (ThermoFisher Cat# 53–9895-82) at room temperature for 30 minutes with constant shaking in the dark. After incubation, 1× agarose loading dye was added, and samples were resolved on a 1% TAE agarose gel alongside a 1 kb DNA ladder (New England BioLabs, MA, USA). Electrophoresis was performed at 80 V for 35 minutes.

Fluorescent imaging was carried out on an Amersham Typhoon Biomolecular Imager (Cytiva, MA, USA) using Cy2 configuration. Following imaging, the gel was stained in 1× PBS (pH 7.4) containing DNA SafeStain (Lamda Biotech, Cat# C138) for 1 hour with gentle rocking and visualized under UV illumination.

### Amino acid deprivation and APEX2 labeling of RNA

Yeast cells were cultured in SC (-Ura) media with 2% D-glucose at 30 °C with vigorous shaking. Cultures were inoculated at an initial OD600 of 0.1 and grown to mid-log phase (OD600 = 0.6–0.7). The culture was divided into two equal volumes and centrifuged at 4,500 × g for 8 minutes at 30 °C to pellet the cells, followed by one wash with 1× PBS and re-centrifugation.

One pellet was resuspended in complete SC (-Ura) medium containing 2% D-glucose, while the other was resuspended in SC (-Ura) medium lacking amino acids but supplemented with 2% D-glucose. Both cultures were incubated at 30 °C with vigorous shaking for 20 minutes, and final OD600 values were measured and recorded.

Cells were then pelleted and resuspended in 500 μL of 1× PBS supplemented with 2.5 mM alkyne-phenol for APEX2-catalyzed RNA tagging, as described above.

### Enrichment of biotinylated RNA using streptavidin beads

Dynabeads MyOne Streptavidin C1 (Invitrogen Cat# 65001) (5 – 7 μg RNA/μL of beads) was equilibrated at room temperature for 20 minutes, washed, and primed for binding according to the manufacturer’s instructions. A total of 100 μg of biotinylated RNA was incubated with the beads for 1 hour at room temperature with gentle rotation. Following incubation, the bead–RNA mixture was placed on a magnetic stand for 3 minutes to allow bead immobilization, and the supernatant was discarded. The beads were then washed again as recommended by the manufacturer.

For elution, beads were resuspended in 50 μL of elution buffer (95% formamide, 10 mM EDTA, pH 8.2, 1.5 mM D-biotin) by gentle flicking until homogeneous. The suspension was heated sequentially at 50 °C for 5 minutes and 90 °C for 5 minutes. Beads were immobilized on a magnetic stand for 3 minutes, and the supernatant containing enriched biotinylated RNA was collected into a fresh tube. The eluted RNA was diluted to <75% formamide with nuclease-free water and purified using the RNA Clean & Concentrator-25 kit (Zymo Research), following the manufacturer’s instructions. The final RNA concentration was quantified using a Tecan Spark multimode plate reader.

### Illumina library preparation and high-throughput sequencing

Between 300 – 1000 ng of total or enriched biotinylated RNA was used for library preparation with the Illumina Stranded mRNA Prep Ligation Kit (Cat# 20040532), following the manufacturer’s instructions. Four biological replicates per condition were processed independently to ensure reproducibility. Final cDNA libraries were quantified with a Qubit 4 Fluorometer (Invitrogen) and assessed for quality and fragment size distribution using an Agilent 4200 TapeStation System (Agilent Technologies) at the Institute of Genomic Medicine (IGM), UC San Diego. Libraries were pooled equimolarly, multiplexed, and sequenced on an Illumina NovaSeq X Plus 10B platform at IGM, generating a minimum of 30 million paired-end reads per sample.

### Sequence Alignment

Base calling and demultiplexing were performed with Illumina BaseSpace^™^ and the DRAGEN^™^ Bio-IT Platform. Sequencing quality was assessed with FastQC (v0.11.9), and all libraries passed Illumina quality control thresholds (Q30, yield). No trimming was required. Reads were aligned to the *Saccharomyces cerevisiae* S288C reference genome (R64–1-1 assembly, Ensembl release 113) using STAR (v2.7.11)^22^. Genome indices were generated with STAR’s --runMode genomeGenerate using the reference genome FASTA (*Saccharomyces_cerevisiae.R64–1-1.dna_sm.toplevel.fa.gz*) and annotation GTF (*Saccharomyces_cerevisiae.R64–1-1.113.gtf.gz*). The --sjdbGTFfile parameter was used to incorporate splice junctions, and --genomeSAindexNbases 10 was specified for the yeast genome. Alignments were performed with 10 threads using the following parameters:

--readFilesCommand zcat

--outSAMtype BAM SortedByCoordinate

--quantMode GeneCounts TranscriptomeSAM

--outFilterMismatchNmax 2

This allowed up to two mismatches per read. BAM files were sorted and indexed with Samtools (v1.13)^23^.

### Read Quantification and Processing

Aligned reads were quantified using featureCounts (v2.0.3, Subread)^24^. Reads overlapping annotated exons of protein-coding genes (Ensembl release 113) were retained. For each condition (enriched and total), per-sample featureCounts tables were merged by gene into a single matrix, column names were standardized, and matrices were checked for consistency across conditions.

### Data Quality Control

Preliminary analyses assessed replicate concordance, outliers, and batch effects to distinguish biological differences between conditions (AA vs. NOAA) and RNA types (total vs. enriched). Count tables were merged by gene and filtered to a curated Ensembl R64 protein-coding set; genes with fewer than 10 total counts across all samples were removed. Sample metadata (condition, batch, RNA type) were joined to the count matrix for DESeq2^25^. Counts were normalized with DESeq2 (median-ratio method) and VST. VST data were used for quality control, including PCA, sample-distance heatmaps with hierarchical clustering, and distribution/dispersion summaries (Figure S1).

### Data Preprocessing

We used the Saccharomyces Genome Database (SGD; R64–5-1) to standardize gene annotations and restricted analyses to verified ORFs (excluding dubious/uncharacterized entries). Processed featureCounts tables for total RNA (T) and translated/enriched RNA (P) were subset to the verified ORF set present in both datasets and aligned to a common gene order. Sample metadata (condition: AA vs. NOAA; RNA type: T vs. P) were consistent across matrices, and the experimental design and sample ordering were validated. anota2seq (v1.30.0) datasets were created from the T and P count matrices with RNA-seq settings. Counts were TMM-log2 normalized, and genes with zero counts across all samples were removed. The differential contrast (AA vs. NOAA) was specified and its direction verified.

### Anota2seq Analysis of Differential Translation

Analyses used anota2seq v1.30.0 with the RNA-seq settings referenced in Larsson et al. (2010)^29^. To screen for concordance during benchmarking, we applied a nominal APV threshold (maxP = 0.05). Reliability safeguards included paired translated/total RNA modeling, random-variance moderation, routine QC, and residual-outlier testing (Figure S4). We required a minimum APV effect size (|log2| ≥ 0.5) and applied predefined slope bounds for translation and buffering/offsetting. Results were generated for four analysis types: translation, buffering, translated mRNA, and total mRNA. Regulatory modes (translation, buffering/offsetting, mRNA abundance) were assigned using anota2seq’s standard classification and summarized with diagnostic plots. For the NOAA vs. AA contrast, candidate genes were selected using the thresholds above across all four analyses. FDR-adjusted p-values (apvRvmPAdj) are reported for each gene.

### Gene Ontology (GO) Analysis

GO enrichment was performed with Metascape (v3.5)^30^ using *S. cerevisiae* ORF IDs with built-in ID conversion. Single-list analyses were conducted for each regulatory mode and direction (translation, buffering, mRNA abundance; up/down). Unless noted, tests were restricted to Biological Process terms, used the whole-genome background, and controlled FDR at 0.10.

For cross-study comparisons (ING, SMR), we used Metascape multi-list enrichment on translation-mode gene sets defined with matched thresholds (|log_2_FC| ≥ 1, p ≤ 0.05), analyzing up-regulated (PSP_Up, ING_Up, SMR_Up) and down-regulated (PSP_Down, ING_Down, SMR_Down) lists separately. Results include per-list and aggregated enrichments with standard term clustering. Genes not handled by Metascape were annotated via SGD.

### Comparative Analysis

We compared PSP results with two reference datasets: polysome profiling^21^ and ribosome profiling^3^. Reported lists of translationally regulated genes were mapped to SGD systematic names and aligned to PSP gene IDs. Gene-level agreement was assessed by overlap between PSP translation-mode calls and the corresponding reference sets, visualized with Venn diagrams generated in JMP (v18.2.2).

Effect-level concordance was performed as referenced in Ingolia et al. (2009)^3^. Up and Down gene sets from each reference were tested for directional shifts in PSP translation APV log2 effects relative to background genes using one-way Mann-Whitney U tests (Up > background; Down < background).

### Quantification and Statistical Analysis

Analyses were performed in R (DESeq2; anota2seq; basic statistics). GO enrichment used Metascape. Tests included one-way Mann-Whitney U test, Pearson correlation, and hierarchical clustering. Venn diagrams for gene-level agreement were generated in JMP.

## Supplementary Material

Supplement 1
