# Supplementary material for: Capturing Translation in Action with Protein Synthesis Profiling
Source: bioRxiv. 2025 Nov 17:2025.11.17.688896. Preprint. [Version 1] doi: 10.1101/2025.11.17.688896 (PMC12667909; doi:10.1101/2025.11.17.688896)
Supplement: Supplement 1 [file media-1.pdf]

Supplementary Information

Capturing Translation in Action with Protein Synthesis Profiling

Cesar Arcasi Matta[1], Zhi Qi Ten[1], and Simpson Joseph*

Department of Chemistry and Biochemistry, University of California at San Diego,
9500 Gilman Drive, La Jolla, CA 92093-0314 USA

1. These authors contributed equally to this work.

* To whom correspondence should be addressed.
Email: sjoseph@ucsd.edu

Supplementary Figures

Supplementary Figure 1: RNA-seq normalization and sample-level quality control.

a, VST mean–SD and expression density plots. Mean–SD (log–log) plots of VST-transformed expression values for total (TOT) and enriched (ENR) libraries in control (CTRL), amino-acid–treated (AA), and NO amino-acid (NOAA) conditions, and density plots of VST expression across all sample groups. The largely mean-independent spread of points and overlapping density curves indicates effective variance stabilization and comparable expression distributions suitable for downstream linear modeling.

b, Boxplots of VST-transformed gene expression values for each sample, grouped by condition (REF, AA, NOAA) and library type (total, T; enriched, E). Similar medians and interquartile ranges across all samples indicate successful normalization and an absence of obvious technical biases or outlier libraries.

c, Principal component analysis (PCA) of VST-transformed expression profiles. Each point represents a sample (REF, blue circles; AA, green squares; NOAA, red triangles). Open and filled symbols indicate total and enriched libraries, respectively. Samples segregate primarily by treatment along PC1 (71% variance explained), with replicate 4 in each treatment displaced toward the edge of its cluster but not forming a separate cluster. This indicates that biological differences, rather than technical artifacts, drive the major variance components.

d, Euclidean distance heatmap of inter- and intra-group sample relationships. Each cell represents the pairwise distance between VST-transformed expression profiles, with hierarchical clustering applied to samples. Replicates cluster tightly within conditions (REF, AA, NOAA) and sequencing types (total vs enriched) and are clearly separated between groups, confirming expected sample-to-sample similarity patterns and overall dataset integrity.

Supplementary Figure 2: anota2seq model diagnostics and signal summaries.

a, Density and frequency plots showing the distribution of raw and Benjamini–Hochberg (BH)–adjusted p-values for the omnibus interaction term in the anota2seq analysis of partial variance (APV) model. The raw p-values follow an approximately uniform distribution with no pronounced peak near zero, indicating that only a minority of genes exhibit interaction effects between treatment and total mRNA levels. The corresponding adjusted p-values cluster near one, supporting the common-slope assumption used by anota2seq.

b, Density and frequency plots showing the distribution of raw and BH-adjusted Random Variance Model (RVM) p-values for the same omnibus interaction term. The approximately uniform distribution of raw RVM p-values suggests that variance moderation preserves the expected null behavior across genes. In contrast, the adjusted p-values cluster tightly near one, indicating that RVM shrinkage stabilizes variance estimates without inflating false positives.

c, Random variance model (RVM) fits for interaction and omnibus group tests. For interaction terms (top) and omnibus group effects (bottom), Q–Q plots (left) compare empirical variance estimates with those expected under the inverse-gamma prior, and cumulative distribution plots (right) overlay empirical (black) and theoretical F-distributions (cyan) with Kolmogorov–Smirnov p-values (0.0191 and 0.0454, respectively). The close correspondence between empirical and theoretical curves indicates that the RVM provides an adequate description of variance across genes.

d, Proportion of outliers in regression assessment using dfbetas. Bar plots show the observed (black) and simulated (red) proportions of data points exceeding several dfbeta cut-offs ($|dfb| > 1$, > 2, > 3, $> 2/\sqrt{N}$, $> 3/\sqrt{N}$, and $> 3.5 \times IQR$). The near-identical proportions indicate no excess of influential observations beyond that expected under a normal-error model, supporting the robustness of the APV regression fits.

e, Density plots of raw p-values for total mRNA, translated mRNA, translational efficiency ("translation"), and buffering analyses in contrast 1. A prominent enrichment of very small p-values for total mRNA and a more modest enrichment for translated mRNA and translation indicate a strong transcript-level and moderate translational signal. In contrast, the flatter buffering distribution is consistent with fewer robust buffering events.

f, Density plots of Benjamini–Hochberg FDR values for total mRNA, translated mRNA, translational efficiency, and buffering analyses in contrast 1. Strong enrichment of low FDRs for total and translated mRNA, a broader distribution for translation, and a peak near 1 for buffering indicate many transcript-level changes, fewer translational events, and relatively few high-confidence buffering events, consistent with the raw p-value distributions.

g, Summary of all residuals from the APV regression model. Vertical bars show empirical residual quantiles across the standard normal distribution, with pink ticks indicating simulated normal envelopes. The expected proportion of outlier residuals is 1%, whereas 0.815% are observed, suggesting that residuals are approximately normally distributed and that the anota2seq model exhibits no excess of outliers.

h, Residuals versus fitted values for the APV regression model. Each point represents a residual from the regression of translated on total mRNA as a function of its fitted value. Residuals form a roughly horizontal band centered around zero with approximately constant spread across the fitted range, supporting assumptions of linearity and homoscedasticity of residuals in the anota2seq model.

Supplementary Figure 3: Metascape GO enrichment of translationally and transcriptionally regulated gene sets from PSP.

a, GO Biological Process enrichment heatmap for translationally repressed (Translation Down) and translationally enhanced (Translation Up) gene sets. Rows correspond to enriched biological processes and the two columns to the down- and up-regulated translation gene lists; colors represent $-\log_{10}(P)$ values. This joint view contrasts functional programs affected in opposite

directions at the level of translation.

b, GO Biological Process enrichment heatmap for transcriptionally repressed (Transcription Down) and transcriptionally enhanced (Transcription Up) gene sets. Rows correspond to enriched biological processes and the two columns to the down- and up-regulated transcriptional gene lists; colors represent $-\log_{10}(P)$ values. This view highlights biological processes that are predominantly governed at the mRNA abundance level, allowing for direct comparison with translational enrichment patterns.

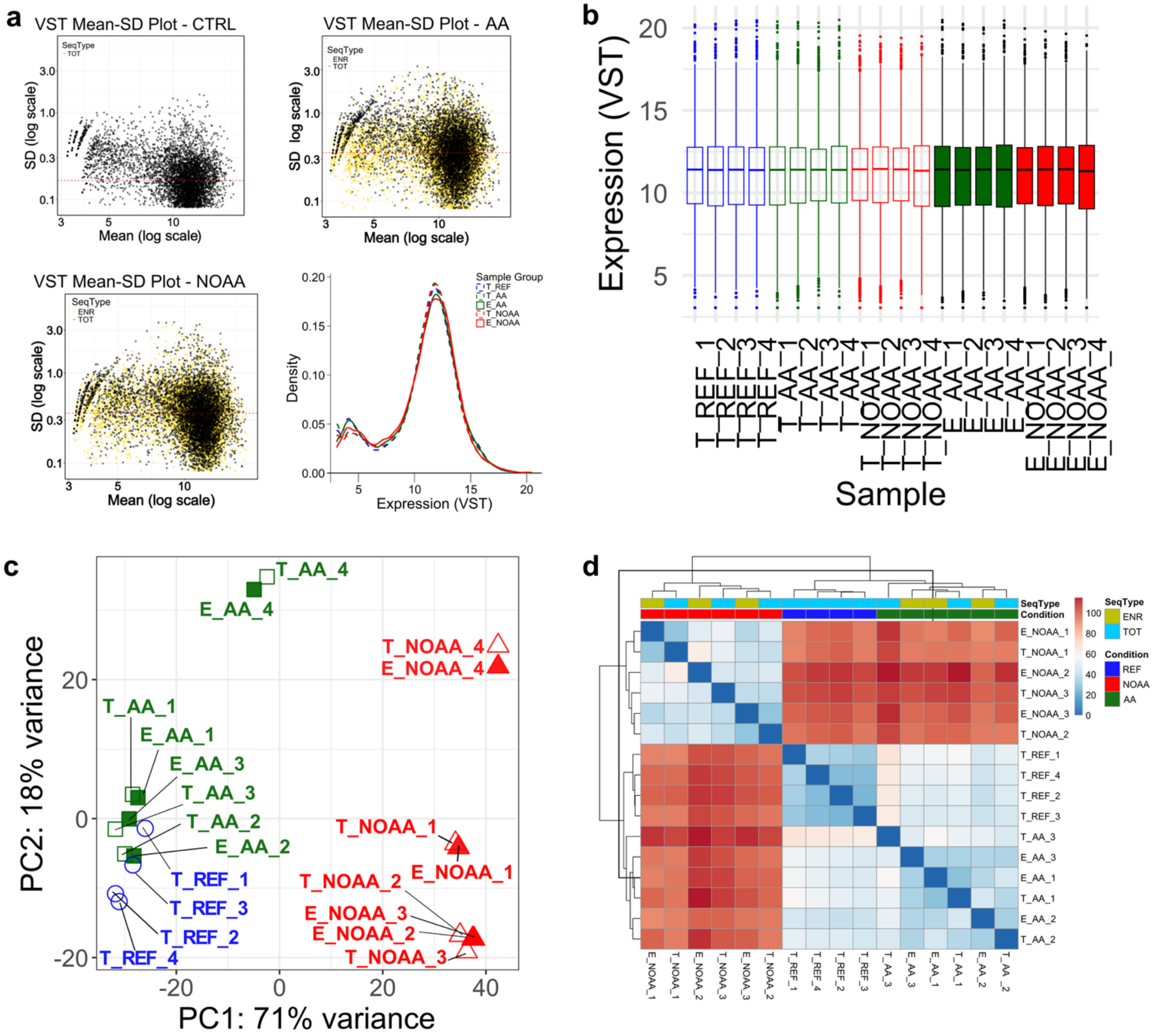

Supplementary Figure 1

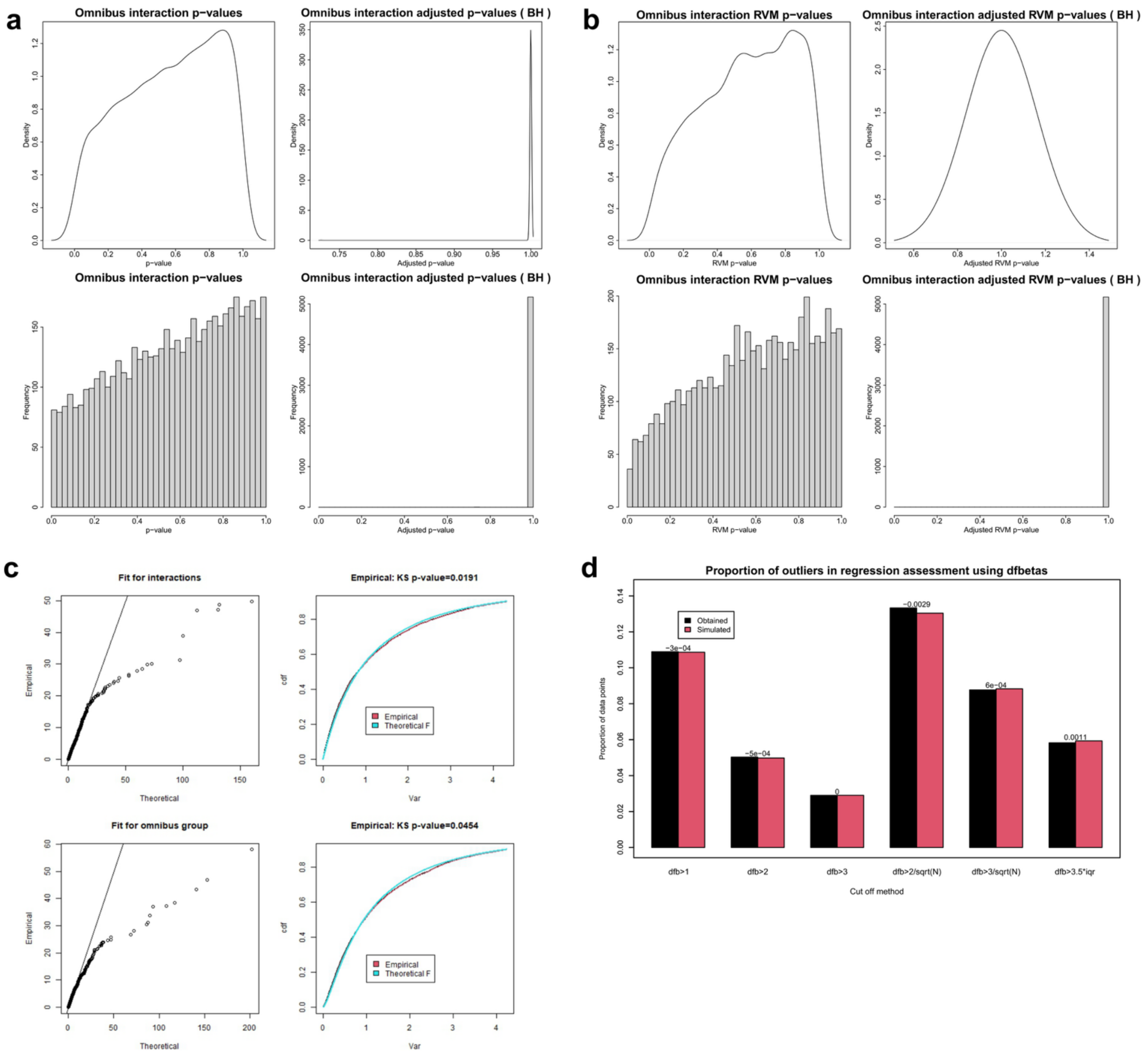

Supplementary Figure 2

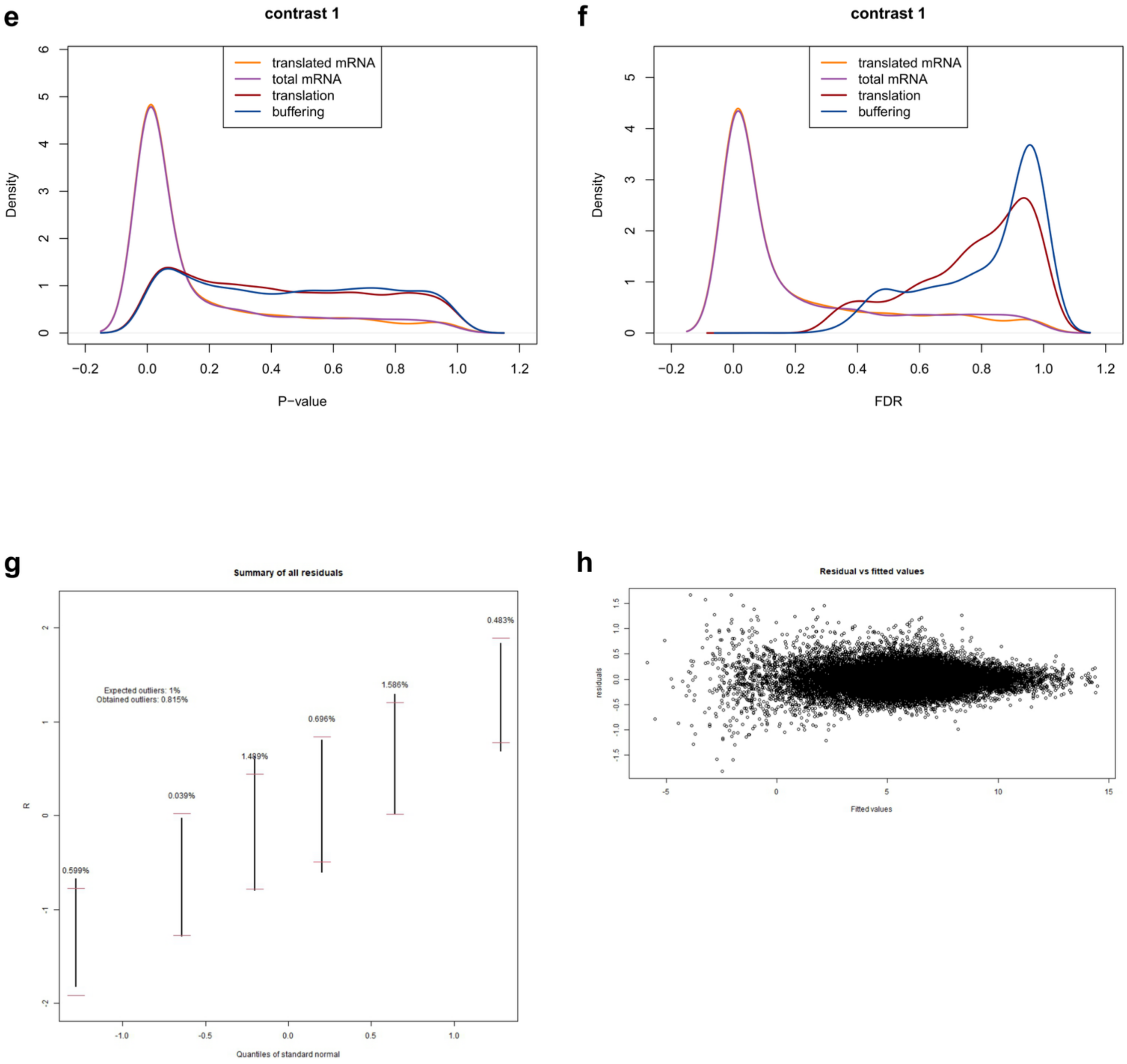

Supplementary Figure 2 (Continued)

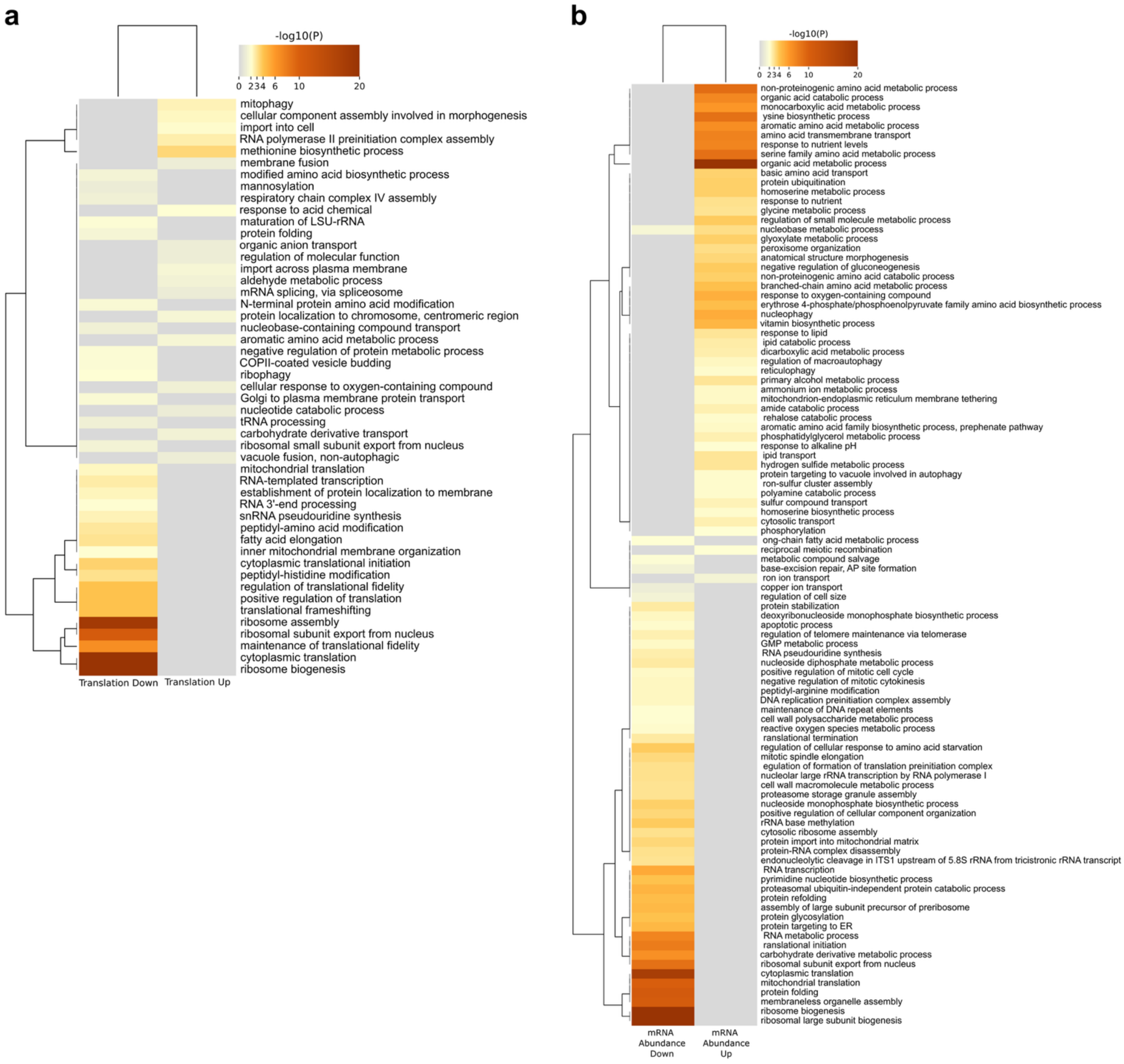

Supplementary Figure 3
